# One Hundred Years of Sulfido Ligand in the Molybdenum Cofactor: New Perspectives

**DOI:** 10.1002/cbic.70527

**Published:** 2026-10-09

**Authors:** Silke Leimkühler, Benjamin R. Duffus, Carola Schulzke

**Affiliations:** ^1^ Institute of Biochemistry and Biology Department of Molecular Enzymology University of Potsdam Potsdam Germany; ^2^ Institute of Biochemistry, Bioinorganic Chemistry University of Greifswald Greifswald Germany

**Keywords:** formate dehydrogenase, molybdenum cofactor, sulfido ligand, superoxide, xanthine oxidase

## Abstract

The xanthine oxidase (XO) family of mononuclear molybdoenzymes consists of well‐studied enzymes like XO, xanthine dehydrogenase (XDH), and aldehyde oxidase (AO) in eukaryotes. The characteristic of this family is a terminal sulfido ligand bound to the active site Mo or W ion. Historically, these enzymes were among the first to be investigated in a purified form. Since its discovery in 1902 in cow's milk, the XO enzyme is among the best‐studied molybdoenzymes. Much pioneering work has been carried out including comprehensive spectroscopic and structural analyses to study the enzymes’ molybdenum center. This led to the discovery that the enzyme produces superoxide and thereby inactivates itself by removal of the essential sulfido ligand at the active site. This sulfido ligand has been later discovered also in enzymes of the dimethyl sulfoxide (DMSO) reductase family. In this review, we describe historical aspects on the discovery and role of the sulfido ligand in the two enzyme families, contextualize new discoveries, and provide perspectives for future investigations into still elusive research questions urgently requiring determination, which may or may not span yet another 125  years.

## Graphical abstract

The terminal sulfido ligand in mononuclear molybdoenzymes has been appreciated for almost 125 years, with the characterization of Schardinger's enzyme in cow's milk, now known to be xanthine oxidase, that was isolated in 1902 in Vienna, Austria. The ligand is found in molybdoenzymes identified across all kingdoms of life. This review highlights the historic and physiological significance the sulfido ligand serves in molybdoenzymes and contextualizes new research questions regarding its involvement.

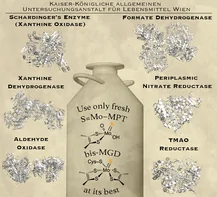

## 1 Introduction: The Families of Molybdoenzymes

Molybdenum has a chemical versatility that is useful to biological systems: It is redox‐active under physiological conditions (ranging between the oxidation states VI and IV); since the V oxidation state is also accessible, the metal can act as a transducer between obligatory two‐electron and one‐electron oxidation–reduction systems, and it can exist over a wide range of redox potentials [1, 2, 3]. The transition element molybdenum is an important trace element for bacteria, archaea, and eukaryotes [4]. In its biologically available form, molybdate (MoO_4_^2−^), it enters the cell by active transport systems [5]. In the cell, molybdenum is either coordinated to the molybdenum cofactors (Moco), which contain a dithiolene group on the 6‐alkyl side chain of a pterin called molybdopterin (MPT) [6] or in some bacteria is part of the Fe–S cluster‐based iron‐molybdenum cofactor (FeMoco) which forms the active site of nitrogenase [4, 7], an enzyme that is not covered in this review.

The biosynthesis of Moco is highly conserved among all kingdoms of life. In all molybdoenzymes containing Moco, the molybdenum atom is coordinated to a dithiolene group of pyranopterin that has been referred to as metal‐binding pterin (MPT) and can coordinate either molybdenum or tungsten; however, two different forms of the cofactor exist: (i) The form found in eukaryotes [8] which contain one dithiolene group (mono‐MPT) ligated to the molybdenum ion, and (ii) the main form found in prokaryotes where two dithiolene pterins (bis‐MPT) are ligated to the molybdenum atom forming the so‐called bis‐metallopterin guanine dinucleotide (MGD)‐Moco [9], most often also containing a guanosine nucleotide at each of the terminal phosphate groups of each pterin.

Importantly, several variations in the ligand sphere of the molybdenum atom or additional modifications at the pyranopterin backbone have been identified. According to these differences within the first coordination sphere of the molybdenum ion, molybdoenzymes have been classified into three families (Figure 1): the xanthine oxidase (XO) family, the sulfite oxidase (SO) family, and the dimethyl sulfoxide (DMSO) reductase family. Newer classifications also include the tungsten‐containing enzymes in one enzyme family [10, 11]. However, the first coordination sphere of tungsten‐containing enzymes is not different from molybdenum‐containing enzymes; therefore, we prefer not to classify tungsten‐containing enzymes into a separate family since most often, in almost all enzymes of the DMSO reductase family, tungsten can be replaced by molybdenum, which changes the reactivity of the enzyme but does not change the first coordination sphere. The original classification was based on the identification of the bis‐MPT cofactor without nucleotides in the crystal structure of AOR, but more recently, in *Escherichia coli*, the YdhV enzyme, a similar cofactor containing molybdenum has been identified, so this classification exclusively for tungsten enzymes is not valid anymore [12]. No tungsten‐containing enzymes have been identified in eukaryotes so far. Enzymes of the XO family contain, in general, a Moco with one MPT (or MCD, metallopterin cytosine dinucleotide) bound to a Mo^VI^OS(OH) moiety, and typical members are xanthine oxidoreductases and aldehyde oxidases (AOs) [2]. In bacteria, variants exist binding the mono‐MPT cofactor with a cytosine nucleotide [12, 13, 14, 15] present at the MPT(MCD), the role of which is to serve mainly as an anchor to bind the cofactor better to the protein backbone [16]. Enzymes of the SO family contain a Moco with a mono‐MPT‐Mo^VI^O_2_Cys core. This family comprises bacterial sulfite dehydrogenases and methionine sulfoxide reductase in addition to SO, nitrate reductase, and mARC enzymes in eukaryotes [17]. No dinucleotide variants of the cofactor have been identified so far to be present in this enzyme family. Enzymes of the DMSO reductase family are exclusively found in prokaryotes [2]. These enzymes comprise a Moco with a bis‐MGD‐Mo^VI^XY core with X being either a sulfur or an oxygen ligand and Y being either a hydroxo or a variable amino acid ligand (Ser, Cys, SeCys, and Asp ligands were identified so far) [18]. Mainly, archaea and some anaerobic bacteria require tungsten (W) instead of molybdenum and coordinate W in a similar manner to the dithiolene group of MPT, forming the tungsten cofactor (Wco) [19, 20, 21]. Here, bis‐MPT‐W^VI^XY cores are found [22]; notably, in *E. coli,* one enzyme with a bis‐MPT‐Mo^VI^‐Cys=S core has been, so far exclusively, identified that binds molybdenum instead of tungsten [11]. While the biosynthesis of Moco is well characterized, the biosynthesis of Wco has not been studied in detail so far. In general, it is believed that the basic mechanisms of Moco and Wco assembly are similar because most genes with an assigned function in Moco biosynthesis are also present in the genome of organisms that use tungsten in their metabolism [23, 24]. Specific enzymes were proposed to insert specifically tungsten into the cofactor backbone; however, the ultimate proof is still elusive, while recent results of *Methylorubrum extoquens* formate dehydrogenase confirm their involvement [25]. For details on molybdenum cofactor biosynthesis, the reader is referred to more specialized reviews on this topic [26, 27, 28].

**FIGURE 1:**
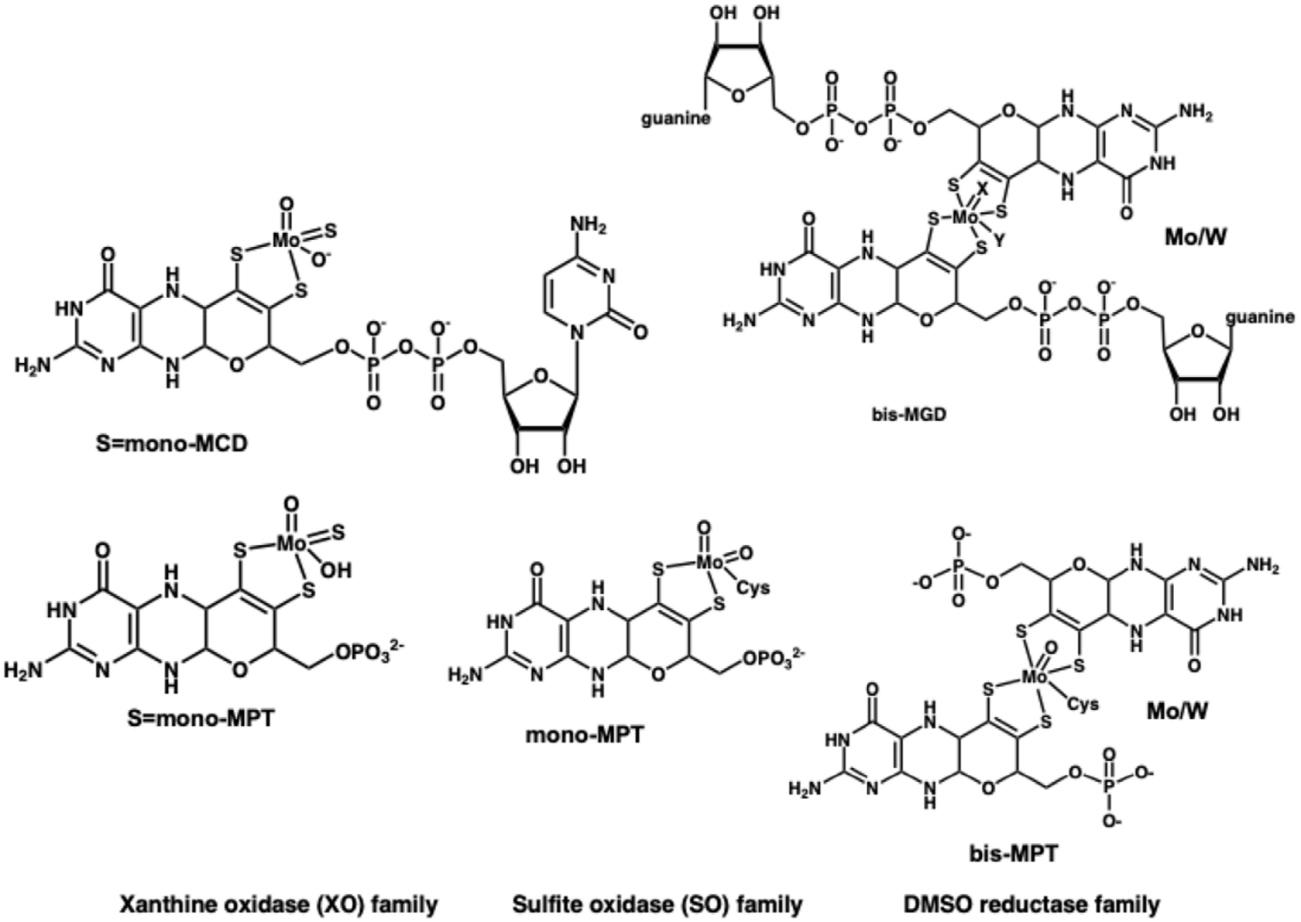
The three families of molybdoenzymes: Details are given in the text.

## 2 The XO Family

The XO family of enzymes catalyze important oxidative transformations of *N*‐heterocycles and aldehydes and are involved in catabolic processes [2, 29], xenobiotic detoxification [30, 31], drug metabolism [32], prodrug activation and conversion [33], and oxidative stress leading to postischemic reperfusion injury [31]. This family generally catalyzes a hydroxylation reaction [2, 34]. This unique type of hydroxylation reaction generates, rather than consumes, reducing equivalents and activates water instead of dioxygen as the source of the oxygen atom inserted into the substrate C—H bond [35].

Xanthine oxidoreductase (XOR) can be found in both oxidase (XO) and dehydrogenase (XDH) forms and has a wide distribution in mammalian tissues, with the highest amounts observed in the intestines and liver [36, 37]. Because the XO and xanthine dehydrogenase (XDH) forms are products of the same gene, the enzyme is best referred to as XOR [2]. Mammalian XOR exists as a homodimer with a molecular weight of about 290 kDa [38]. Bovine XOR is the prototypical member of this family and is one of the longest‐studied enzymes, having been first purified to homogeneity in 1902 from bovine milk and later from recombinant bacterial systems, first designated as the Schardinger enzyme [39, 40]. The enzyme as typically isolated from cow's milk is an oxidase, using O_2_ as an electron acceptor, but the form expressed in most vertebrate tissues is first the dehydrogenase form that utilizes preferentially NAD^+^ rather than O_2_ [41]. The mammalian dehydrogenase is reversibly converted to the oxidase by oxidation of thiols to disulfides or irreversibly converted by mild proteolysis [42]. XOR is present in milk and has, in fact, been detected in mammary gland epithelial and capillary endothelial cells [43]. Intracellularly, XDH is the predominant species, while XO is present in the gastrointestinal lumen and body fluids. XO has been shown to be a potent generator of reactive oxygen species (ROS). In ischemic tissues, the enzyme converts to XO and acts on accumulated hypoxanthine during reperfusion, producing a massive, tissue‐damaging burst of superoxide and hydrogen peroxide [44]. Milk is exceptionally rich in XO, where it might act as a natural antimicrobial agent based on the production of ROS [43, 45]. However, inactive forms of XOR do occur in mammalian tissues and particularly in milk and usually lack molybdenum or sulfur in the Moco cofactor. A structural role of XO has been assigned in milk droplet formation, thus explaining why inactive forms of XO are present and still beneficial in milk [46, 47, 48]. It was estimated that up to 60% of XOR found in bovine milk may be in an inactive state [43, 49, 50]. Thereby, the demolybdo XOR lacks the molybdenum (Mo) and possibly the whole MPT cofactor, while the desulfo XOR has a molybdenum‐bound sulfur atom replaced by oxygen in the Moco (see below) [31, 51].

### 2.1 Identification of the Cyanolysable Sulfur

Active XOR can be converted to an inactive enzyme by treatment with cyanide or, more slowly, but inevitably, by storage and/or superoxide exposure [52]. The inactivation of the enzyme with cyanide was first reported by Dixon and Thurlow [53] and reinvestigated by Dixon and Keilin [54] since some controversies existed. Dixon and Keilin reported that the rate of inactivation is dependent on the cyanide concentration, the temperature, and, importantly, the order of the addition of the components. The best temperature for inactivation was observed to be 45 °C. Cyanide inhibition is more pronounced at higher cyanide concentrations. It was found to be very important that cyanide needed to be added first before hypoxanthine was added to the assay for activity measurements; otherwise, hypoxanthine would protect the active site and prevent cyanide from reacting with it. Thus, preincubation with cyanide gave the best inactivation results. In their studies, the mode of cyanide inactivation on the enzyme was not clear. Later investigations by Rajagopalan and Coughlan showed that upon cyanide treatment, XO is converted to the inactive desulfo‐form, in which the sulfido ligand is replaced by an oxido ligand, and SCN^−^ is released [52]. The fact that cyanide treatment of XOR (and related enzymes, like AO but not SO) resulted in inactivation and release of SCN^−^ has been known since 1970, and the nature of the cyanolysable sulfur has been the subject of extensive speculation [55]. Williams and Wentworth first proposed in 1974 that this sulfur was linked to molybdenum as a terminal sulfido group [56].

In 1958, Fridovich and Handler reported that incubation with ^14^C‐labeled NaCN resulted in the tight binding of ^14^CN to the inactivated enzyme with a stoichiometry of 1 molecular equivalent (eq) of ^14^CN bound per eq of enzyme flavin [57]. They also reported that dithionite prevented both the inactivation and fixation of ^14^CN. Later, Rajagopalan, Handler, and Coughlan and Rajagopalan and Handler have reported similar results with XO and two other molybdenum‐containing iron–sulfur flavoproteins, AO and XDH [52, 58]. These researchers concluded that cyanide inactivation is due to cyanide forming a complex with the enzyme‐bound molybdenum, thus preventing substrate binding and electron transfer through the sequence of molybdenum, flavin, and iron–sulfur chromophores. The existence of a molybdenum–cyanide complex was, however, inconsistent with the failure to observe an isotope effect on the molybdenum electron paramagnetic resonance signals of cyanide‐inactivated XO relative to the aforementioned radiolabel study [59].

It was later confirmed that upon cyanide treatment of the enzyme, SCN^−^ is released, and “desulfo” enzymes are formed that have no catalytic activity [60]. The proof for the loss of the sulfido ligand upon cyanide treatment was obtained by EXAFS studies 1980 of the cyanolysed enzyme in comparison to the active enzyme and to SO [61, 62, 63, 64, 65]. The EXAFS results of the molybdenum site for cyanolysed XDH are quite consistent with the structure predicted for the intact enzyme, suggesting that the major effect of cyanide treatment can be viewed as the replacement of a terminal sulfido ligand with a terminal oxido group. It was interesting that no further change was observed in the bond lengths of the remaining sulfur ligands from the dithiolene group and that they therefore remained about 0.05 Å longer than the corresponding values obtained from SO, regardless of the nature of the terminal chalcogen ligand [66].

In addition, more than 100 years ago, Dixon (1925) showed that the incubation of bovine milk XO with hydrogen peroxide resulted in the inactivation of the enzyme [67]. This observation has since been confirmed several times with XO and with the related XDH [68, 69]. Apart from nonspecific modes of inactivation, the various prosthetic groups in these enzymes have been suggested to be the targets for attack by hydrogen peroxide [70, 71]. However, the nature of the reaction involved had not been investigated in detail back then, nor has the relation between the sites of inactivation and catalysis been clearly established. It had, however, been concluded in 1979 that H_2_O_2_ treatment of the enzyme likely results in the desulfo form at the molybdenum active site [69].

Only more recently in 2021 and 2023, it was reported for AO that the sulfido ligand is removed upon exposure of the substrate‐reduced enzyme to superoxide and hydrogen peroxide, and no other site of the enzyme has been damaged by these ROS species [72, 73]. This inactivation of an enzyme by hydrogen peroxide and superoxide resulting in removal of the Mo=S sulfido ligand was recently also reported for other enzymes like formate dehydrogenase [74] (Figure 2B) (see Section 3).

**FIGURE 2:**
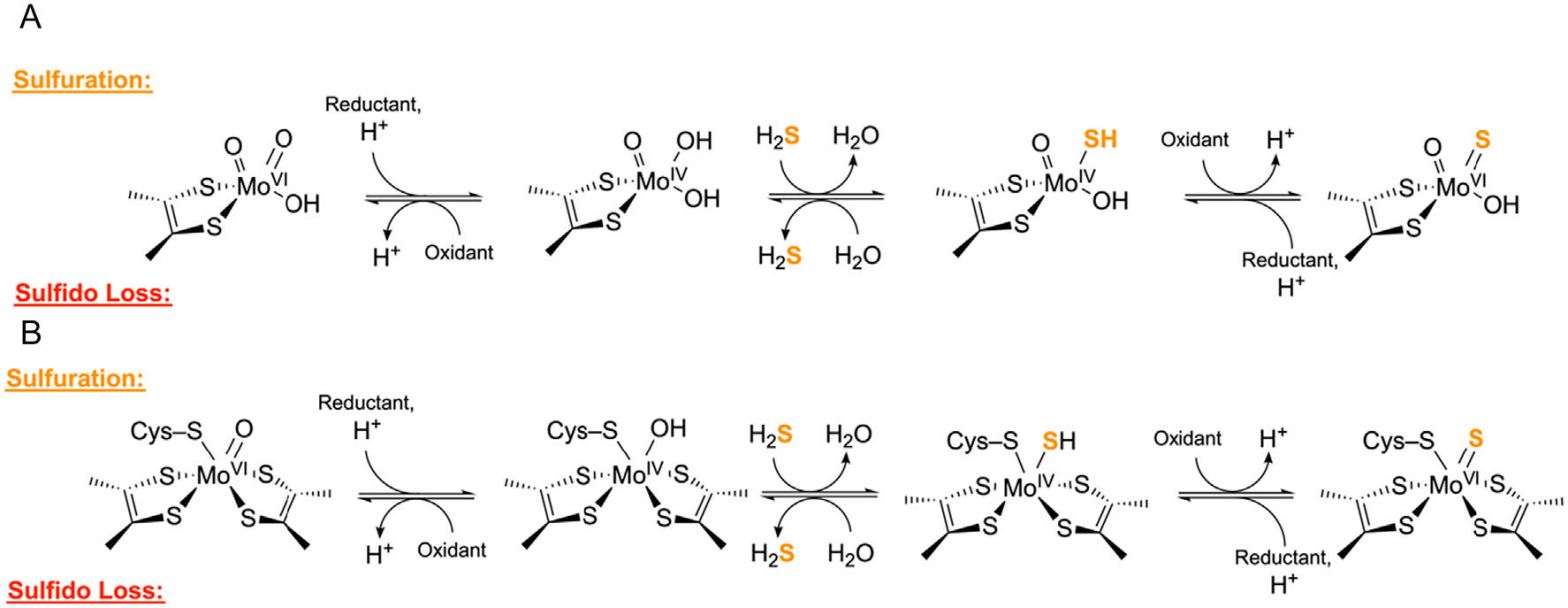
Mechanism of in vitro sulfuration and sulfido ligand loss of the mono‐MPT cofactor in XO family enzymes (A) and the bis‐MGD cofactor in FDH enzymes (B). In vitro sulfuration occurs via reduction of the catalytically incompetent oxido‐coordinating cofactor, that results in a Mo—OH bond that is formed. Sulfide that is supplied or generated in situ replaces the Mo—OH, forming a Mo—SH bond which, following subsequent oxidation of the Mo metal ion, forms a Mo=S bond. Loss of the sulfido ligand is thought to occur in a similar fashion, in reverse, by which reduction and exchange of the sulfido ligand due to the presence of peroxide or superoxide can replace the bound sulfide with a hydroxo ligand that upon oxidation forms the oxido ligand.

### 2.2 Reactivation of XO Activity

As it was evident that cyanide inactivation was due to the elimination of sulfur from the protein as thiocyanate, it was of interest to determine whether the sulfur could be reintroduced, resulting in the return of activity. It was found by Massey and Edmondson in 1970 that incubation with Na_2_S would accomplish this under suitable conditions [55]. The degree and speed of reactivation depended on the pH and temperature. Maximal reactivation was obtained with reproducibility by incubation with 10 mM Na_2_S at 45 °C and pH 9.0.

Most often, a 100% resulfuration of the enzyme could not be realized, based on the fact that inactive species were obtained by replacement also of the other oxido ligands at the active site, multiple sulfido replacements, formation of persulfides at the active site, or simply because nonoptimal conditions were applied during the resulfuration treatment [55, 75, 76, 77, 78].

### 2.3 Role of the Sulfido Ligand in the XO

XO is the prototypical member of the molybdenum hydroxylase family of enzymes [1, 34]. It can catalyze the hydroxylation of purines in the 2, 6, and 8 ring positions (e.g., purine to hypoxanthine to xanthine to uric acid), as well as the oxidation of a wide range of aldehydes and other substrates, although its physiological role is thought to be in purine oxidation [31, 78, 79]. Substrate oxidation occurs at the molybdenum site, which becomes reduced from Mo^VI^ to Mo^IV^ in the process. Detailed structural information on the active site of XO is available from both protein crystallography and spectroscopy, and this has provided valuable insights into the catalytic mechanism [78, 80, 81]. It is now generally agreed that the active site molybdenum atom is ligated by one short terminal sulfido unit (Mo=S), two long thiolate ligands (Mo—S) from the dithiolene, and one short terminal oxygen (Mo—O) [82, 83]. Two major controversies about the active site structure remained, however, unresolved for a couple of years (1996–2004): the position of the Mo=S group in the molybdenum coordination sphere and the nature of the catalytically labile oxygen. With regard to the position of the Mo=S group, the initial crystallographic studies of AO in 1995 and related enzymes placed this ligand in the apical position of a distorted square‐pyramidal coordination geometry for the enzyme in complex with a mechanism‐based inhibitor [76, 84]. In the reduced enzyme, the Mo=S group, which is present in the oxidized enzyme, is transformed to Mo—SH by protonation, which accompanies the reduction. A later study in 2004, however, clearly identified O in the apical position, with the Mo—SH of the reduced enzyme (derived from Mo=S) in the equatorial plane [78]. The initial catalytic mechanism of XO suggested the catalytically labile oxygen site as Mo—OH rather than Mo—OH_2_. From a chemical standpoint, the base‐catalyzed deprotonation of Mo—OH to Mo—O, rather than deprotonation of Mo—OH_2_ to Mo—OH, is expected to yield a significantly better nucleophile that can more effectively initiate the catalytic sequence by attacking the C‐8 position of the substrate [85]. The ultimate proof that the Mo=S ligand is in the equatorial and not the apical position, as also suggested by magnetic circular dichroism spectroscopic analysis in 1999 [86], was finally obtained in 2004 from a high‐resolution crystal structure of XOR [78]. Determining the orientation of the sulfido ligand in the structure was extremely important since this is a catalytically essential ligand. In the reduced Mo^IV^ state, the terminal sulfido ligand is protonated and coordinated to Mo as a sulfhydryl (—SH) group.

Also essential for catalysis is a conserved glutamate (Glu) that functions as a base to activate the Mo—OH hydroxide for nucleophilic attack on substrates. Mo K‐edge EXAFS of oxidized XO showed that the Mo—O distance decreases from 1.97 Å at pH 6 to 1.75 Å at pH 10, and this was consistent with metal‐activated water being present as a hydroxide ligand at physiological pH [87, 88]. The EXAFS work provided strong evidence that this ligand was not a water molecule, as had been previously suggested by earlier X‐ray crystallographic studies, and supported a reaction mechanism that begins with a base‐assisted nucleophilic attack of the substrate by Mo—O(H) [89]. A second Glu is found in the substrate‐binding pocket that may be involved in controlling the tautomerization state of the substrate, while an active site arginine (Arg) has been proposed to affect the orientation of substrate molecules [90]. These Glu and Arg residues located in the substrate‐binding pocket of XOs are not conserved in AOs [91].

In summary, the catalytically essential sulfido ligand in XO and AO enzymes plays a critical role in the enzyme mechanism by functioning as a formal hydride acceptor in the heterolytic cleavage of substrate C—H bonds. The formal hydride transfer (consisting of 1 H^+^ and 2 e^−^) from the substrate to the *cis*‐[MoOS] unit results in the two‐electron reduction of the Mo center and protonation of the terminal sulfido to yield a Mo^IV^—SH center. Thus, the closed shell nature of the hydroxylation mechanism is decidedly different from mechanisms proposed for the distinct group of monooxygenase enzymes in general, which use molecular oxygen as an oxygen donor instead of water as in the molybdoenzymes.

### 2.4 Role of ROS Production by XO in Cancer

As another cellular involvement, XOR has been implicated in cancer, in particular with regard to aggressive forms of breast cancer. To analyze whether the connection with cancer is based on the ROS production mainly produced by the XO form, the group of T. Nishino in Japan has produced a XO *knock‐in* mouse that only contains the oxidase form and a dehydrogenase *knock‐in* mouse that only contains the dehydrogenase form for comparison [92]. The results and thorough investigation of these two mice groups showed that in vitro, XO *ki* macrophages produced higher levels of ROS responsible for an increased regulatory T‐cell (Treg) level observed in the tumors, which are important for the adaptive immune system. The group had speculated that higher ROS production in XO *ki* macrophages could lead to increased Treg differentiation. Tumors with high Treg/T cell ratios were favorable for tumor growth and predicted poor cancer patient survival. Blocking ROS in vivo slowed down the tumor growth. Collectively, these results indicated that the conversion of XO/XDH plays an important role in immune surveillance of tumor development and that the conversion of XDH to XO is important to balance for the prevention of breast cancer [93].

In their study, strikingly, ROS had a dose‐dependent effect on T cells. At low concentrations, ROS are required for effector T cell differentiation; at higher levels, they down‐regulate T cell activity and switch the immune response toward Treg phenotype, and, at very high concentrations, they become cytotoxic. Indeed, the Nishino group found an increase in Tregs in the fastly growing tumors in XO *ki* mice compared to those in XDH *ki* or WT mice [92]. Also, in vitro, XO *ki* macrophages induced much higher Treg differentiation from native T cells. Blocking ROS in vitro and in vivo resulted in reduced Treg formation and reduced cancer growth, respectively.

As explained above, it was shown that ROS production by XO and AO during catalytic turnover of the enzyme resulted in an inactivation of the enzyme by removal of the sulfido group based on the produced superoxide and hydrogen peroxide at the enzyme flavin site [72, 73]. Thus, it needs to be investigated in future studies if the dose‐dependent effect of ROS produced by XO is used to limit ROS production by a feed‐back‐inhibitory effect of ROS, inactivating the enzyme that produces the ROS. This self‐regulatory mechanism might be important for cancer prevention.

## 3 Is the Sulfido Ligand Also Common in Bis‐MGD Enzymes?

### 3.1 Expansion of the Sulfido Ligand to the Bis‐MGD Cofactor Present in Prokaryotes

The first crystal structures of enzymes to be solved from the DMSO reductase family were those of *R. capsulatus* DMSO reductase in 1996 [93, 94] and *E. coli* formate dehydrogenase in 1997 [95].

The first evidence to support the presence of a sulfido ligand at the active site of a DMSO reductase family enzyme constituted the intersection of several pieces of experimental evidence. The X‐ray structure of DMSO reductase revealed the presence of the bis‐MGD cofactor in this enzyme family [93, 94]. However, three different structures of the bis‐MGD cofactor were identified from three different groups [96, 97], and the overall structure was finally resolved to represent two oxo groups in addition to the serine ligand and the two dithiolene groups bound to the active site Mo ion [98].

In contrast, assessment of the *E. coli* formate dehydrogenase structure in relation to spectroscopic data provided the basis for the presence of a sulfido ligand. The structure of the FdhF enzyme from *E. coli* was originally solved in the oxidized then formate‐reduced state and in the oxidized state in the presence of an inhibitor anion, nitrite [95]. While the Mo^V^ state of *E. coli* FdhF was shown to represent a Mo ion coordinated with the amino acid ligabound [99, 100], different data exist on the nature of the reduced Mo^IV^ state. In a reinterpretation of the crystallographic data of the formate‐reduced *E. coli* FdhF enzyme, it was suggested that the selenocysteine residue was dissociated from the Mo ion and shifted 12 Å away [101]. In addition, the reanalysis showed that a sulfido ligand had replaced the oxo ligand. It is now assumed that the sulfido ligand was lost in the *E. coli* FdhF enzyme based on the purification/crystallization and storage conditions that have to be strictly anaerobic. This sulfido ligand was later identified in a high‐resolution X‐ray crystal structure of the W‐containing isoenzyme of formylmethanofuran dehydrogenase from *Methanoanaerobacter wolfeii* [102]. The presence of the sulfido ligand in FDH enzymes was further confirmed in *R. capsulatus* FDH by EXAFS studies and confirmed in *C. necator* FDH by EPR studies [103, 104]. In a recent X‐ray absorption spectroscopy (XAS) study on *R. capsulatus* FDH, it was shown that the sulfido ligand was lost, forming thiocyanate (SCN^−^) upon treatment with cyanide, which represented another similarity with XO [105]. The resulting desulfo‐enzyme harbors an oxo‐group in place of the sulfido group that is not able to act as a hydride acceptor. The first example of cyanolysis of an FDH enzyme was reported for *Methanobacterium formicicum* FDH in 1983 that resulted in a loss of activity and an altered Mo^V^ species, reflecting modification of the bis‐MGD cofactor and a shifted redox potential [106] (Table 1). However, examples of FDHs that exhibit apparent or partial CN^−^ insensitivity are known, including W‐FDHs from *Desulfovibrio desulfuricans* [111, 112] and *Megadesulfovibrio gigas* (formerly *Desulfovibrio gigas*) [113] as well as the W‐containing isoenzyme of formylmethanofuran dehydrogenase from *Methanoanaerobacter marburgensis* [114].

**TABLE 1:** Mo redox potentials of molybdoenzymes with sulfido and oxido‐containing active sites.

	Mo species	*E*_m_ (mV vs. SHE)	Titration pH	References
Sulfido				
*M. formicicum* FDH	Mo^VI^–Mo^V^	−330	7.7	[106]
	Mo^V^–Mo^IV^	−470		
*C. necator* FDH (FdsDABG)	Mo^VI^–Mo^V^	−310	7.5	[107]
	Mo^V^–Mo^IV^	−470		
*R. capsulatus* FDH	Mo^VI^–Mo^V^	−408	7.5	[108]
	Mo^V^–Mo^IV^	−477		
Bovine milk XO (rapid)	Mo^VI^–Mo^V^	−355	8.2	[109]
	Mo^V^–Mo^IV^	−355		
Chicken liver XDH (rapid)	Mo^VI^–Mo^V^	−357	7.8	[110]
	Mo^V^–Mo^IV^	−337		
Oxido				
*M. formicicum* FDH	Mo^VI^–Mo^V^	−200	7.7	[106]
	Mo^V^–Mo^IV^	−210		
Bovine milk XO (slow)	Mo^VI^–Mo^V^	−440	8.2	[109]
	Mo^V^–Mo^IV^	−480		
Chicken liver XDH (slow)	Mo^VI^–Mo^V^	−397	7.8	[110]
	Mo^V^–Mo^IV^	−433		

Crystallographically, a sulfido ligand on a bis‐MGD cofactor was first detected in formate dehydrogenase from *M. gigas*. The structure upon refinement resulted in a Mo–S distance of 2.39 Å without positive residual density, suggesting that an additional sulfido ligand was present [115, 116]. This observation was corroborated by subsequent reanalysis of the formate dehydrogenase subcomplex structure [117] and the periplasmic nitrate reductase structure [118], the latter having been further supported with additional structures solved from *D. desulfuricans* and *C. necator* H16 [119, 120]. The long Mo—S distance and the close positioning of the active site SeCys with the sulfido ligand appeared to suggest that the sulfido was a bound persulfide between the active site metal and amino acid [119]. However, subsequent X‐ray absorption spectroscopic measurements on *R. capsulatus* FDH with an optimized sulfido ligand content showed a shorter Mo—S distance of 2.25 Å, reflecting a bound terminal Mo=S bond [105]. Collectively, these observations reflect a dynamic role played by the sulfido ligand, which points to an active engagement in a redox‐associated hydride‐transfer reaction.

Following the detection of a bound sulfido ligand in FDH, attempts to account for the functional role of the sulfido ligand in the DMSO reductase family have been made. For FDH, it had been largely accepted to involve hydride transfer in a fashion similar to XOR/AOX [104]. One qualitative difference between XOR/AOX and FDH is that the latter coordinates an additional amino acid residue with the metal ion [2]. An apparent discrepancy regarding the function of the sulfido ligand in relation to the amino acid ligand has persisted because in the presence of the two ligands, the coordination sphere of the central metal ion is saturated and no binding site is available for H_2_O needed to support an oxygen atom‐transfer (OAT) step that is observed for XOR/AOX. To date, no crystallographic structures have been determined that clearly show H_2_O coordination displacing only the bound Cys/SeCys without sulfido ligand loss; thus, it has been proposed that the catalytic reaction can occur exclusively in the secondary coordination sphere, without Cys/SeCys binding/unbinding [104, 121, 122, 123].

A coordinated sulfido ligand has also been suggested in the Mo coordination sphere of periplasmic nitrate reductases and in the *E. coli* TMAO reductase, but its presence as a bound ligand in these enzymes is a current item of debate. A comparison of the pros and cons of how the enzymes were purified in relation to sulfido‐ligand‐containing FDHs is provided in Table 2. We emphasize that careful consideration of the methods for isolating and expressing the enzyme critically impacts the retention of the sulfido ligand at the active site. Similarly, detection methods like cyanolysis likely cannot be applied to all enzymes, as reflected by the insensitivity of this method to some FDH enzymes. It has to be considered that some inhibitors like CN^−^ might not be accessible to the active site of nitrate reductase or TMAO reductase since it is not a substrate analogue for these enzymes.

**TABLE 2:** Comparison of selected DMSO reductase family enzymes and associated experimental methods to support the presence or absence of a coordinated active site sulfido ligand.

Enzyme	Purification conditions	Kinetically active?	Sulfido ligand?	Methods to detect sulfido	Comments	Literature references
*E. coli* TMAO reductase	Anaerobically/aerobically	Yes/no	Yes/no	Electrochemistry, −0.23 V = O; −0.45 V = S	Inactive + O_2_, Active − O_2_; sulfido ligand O_2_‐sensitive	[124]
*E. coli* TMAO reductase	Aerobically	No	No	XAS	Inactive upon aerobic purification; active upon dithionite‐based redox‐cycling; dithionite known sulfido source	[125, 126]
*C. necator* periplasmic nitrate reductase	Aerobically (periplasm)	?	Yes (persulfide)	1.5 Å X‐ray crystal structure	Isolated from periplasm, no oxo ligand assignable upon single wavelength anomalous diffraction	[120, 127]
*D. desulfuricans* periplasmic nitrate reductase	Aerobically (periplasm)	?	Yes (persulfide)	X‐ray crystal structure (single wavelength anomalous diffraction), EPR	Isolated from periplasm, initially purported oxo ligand identified as sulfido upon single wavelength anomalous diffraction	[119, 128]
*C. jejuni* periplasmic nitrate reductase	Aerobically (overexpression)	Yes (low)	No	Sörbo, XAS, EPR	Aerobic purification and handling perhaps lost the sulfido; 100% oxo proven, but of low apparent activity, no SCN^−^ detectable	[129, 130, 131, 132]
*C. necator* formate dehydrogenase (Fds)	Aerobic, nitrate‐inhibited	Yes	Yes (persulfide)	EPR, XAS	Sulfido ligand assigned by EPR later assigned to be persulfide; oxidation state not clear, claimed that Mo(IV) should lose bound sulfur ligand	[74, 104, 121, 133]
*R. capsulatus* formate dehydrogenase	Aerobic, azide‐inhibited	Yes	Yes (sulfido)	EPR, XAS, Cryo‐EM structure	Sulfido proven by several methods in the presence of azide; lack of inhibitor results in sulfido loss, sulfido can be chemically reinserted	[103, 105, 134, 135]

While metal‐dependent FDH enzymes have been studied for several decades and the *E. coli* FdhF enzyme was among the first molybdoenzymes to be crystalized [95], details of the reaction mechanism involving the first and second coordination sphere remain poorly understood, and the catalytic mechanism of formate oxidation is still unclear [136, 137]. Overall, on the basis of different experimental data and theoretical calculations, different catalytic mechanisms for FDHs have been proposed. Proposed mechanisms relevant to the current state of research are depicted in Figure 3 for comparison. So far, none of these mechanisms is clearly proven or accepted in the field, and aspects of them are highly under current debate.

**FIGURE 3:**
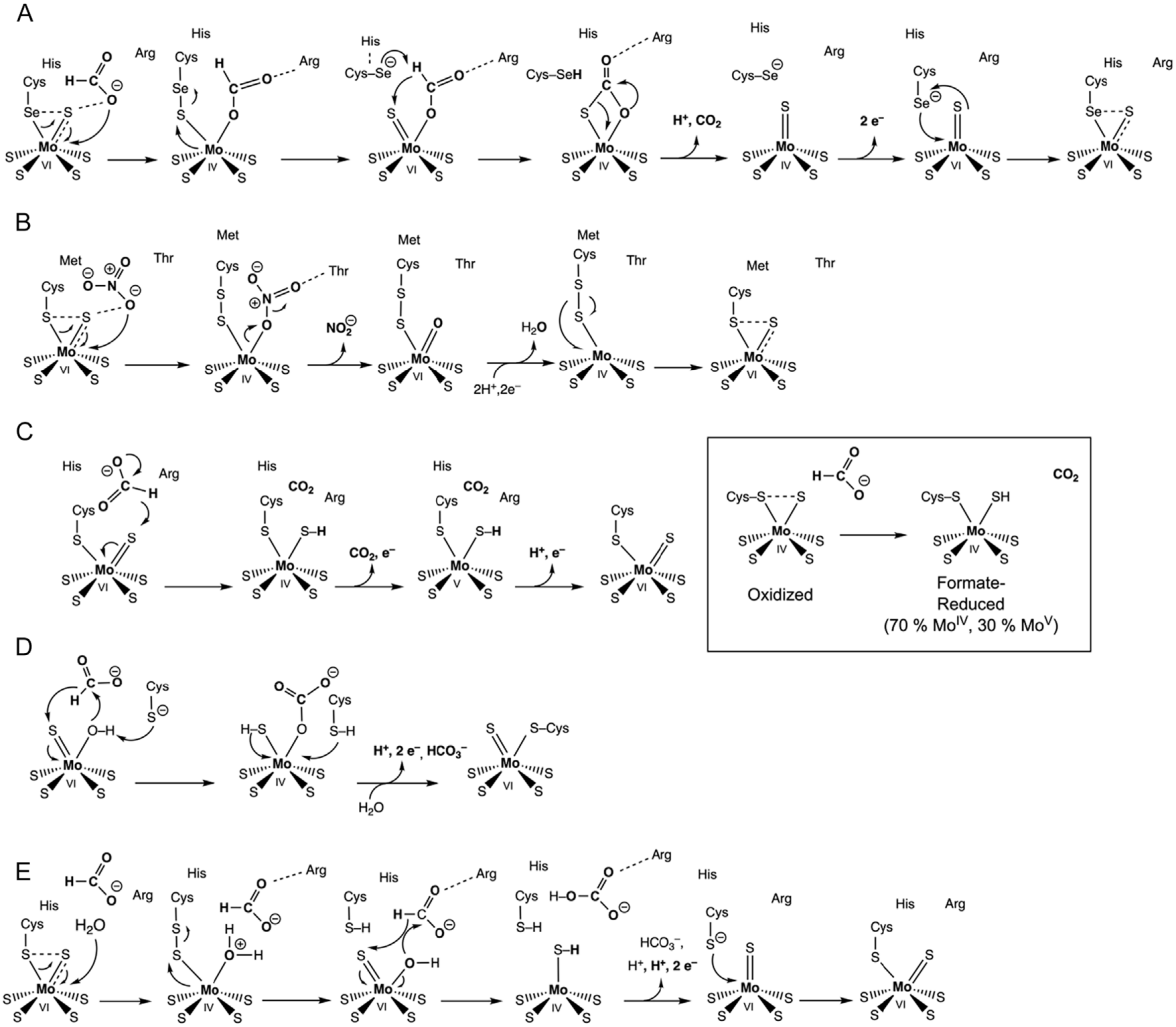
Current running proposals on the chemical mechanism of formate oxidation catalyzed by metal‐dependent formate dehydrogenases, as described in the text that follows. (A) depicts the formate dehydrogenase mechanism inspired from the “sulfur shift” mechanism proposed by Cerqueira, Gonzalez, and Moura for *E. coli* FdhF coordinating a selenocysteine ligand. (B) depicts for reference the sulfur shift mechanism for periplasmic nitrate reductase that was proposed by Cerqueira, Gonzalez, and Moura. (C) depicts the formate oxidation mechanism of *C. necator* FDH proposed by Hille and Niks. Recent revisions to their mechanism regarding the Mo coordination environment based upon recent XAS measurements is depicted in the paneled box. (D) depicts the FDH formate oxidation mechanism of *R. capsulatus* FDH proposed by Kumar and coworkers, featuring active‐site cysteine ligand binding and unbinding. (E) depicts a revised mechanism of the mechanism proposed in (D) that takes into consideration observations noted in respective proposed mechanisms.

A central question is whether the amino acid ligand (selenocysteine or cysteine) remains coordinated to the Mo or W metal during catalysis or whether it dissociates to provide a vacant site for substrate binding. This aspect directly influences the role that the sulfido ligand serves mechanistically with the substrate. An approach to ascertain the function of the sulfido ligand has been to address the lability of the active‐site amino acid bound to the Mo or W ion via inhibition studies with iodoacetamide, an alkylating agent that reacts with “free” thiols. For both the *E. coli* and the *R. capsulatus* enzymes, an alkylation of the selenocysteine or cysteine ligand was observed in the formate‐reduced and NO_3_^−^/N_3_^−^ inhibited enzymes but not in the oxidized enzyme [138, 139]. It should be noted that the presence of NO_3_^−^ or N_3_^−^ is known to protect the sulfido ligand; thus, the apparent inactivation of FDH could be assigned to lack of coordination of the active site Cys/SeCys ligand, as opposed to sulfido ligand loss. However, no alkylation of the selenocysteine residue could be identified in the W‐containing enzyme from *Nitratidesulfovibrio vulgaris* Hildenborough (formerly *Desulfovibrio vulgaris* Hildenborough) [140]. The *E. coli* FdnGHI enzyme likewise was not alkylated when incubated with formate [141]. Collectively, IAA FDH inactivation has shown that the active site Cys/SeCys residue is catalytically essential.

Regarding whether the amino acid ligand (selenocysteine or cysteine) remains coordinated to or becomes dissociated from the Mo or W metal during catalysis, the numerous mechanisms that have been proposed for FDH reflect a lack of clarity of the coordination environment and oxidation state of the metal at the bis‐MGD cofactor. Currently, it is assumed that this reaction is not an OAT reaction, as characteristic of many Mo/W‐bis‐MGD family enzymes. It has been proposed that the immediate reaction product is rather CO_2_ than bicarbonate (HCO_3_^−^), the product expected in an OAT mechanism [142, 143, 144]. However, this is also under debate since in a recent NMR study using thioformate as a substrate analogue, the immediate product to be detected was thiocarbonate, before carbonyl sulfide was produced in a much slower secondary reaction [145]. Further, doubly labeled ^13^C^18^O^16^O was detected in two studies using ^13^C‐labeled bicarbonate and ^18^O‐labeled water, albeit with different interpretations and different experimental setups [100, 122, 145]. It, however, became obvious that the catalytic mechanism has to be studied in the absence of any inhibitor present since the inhibitor results in an interference with the reaction mechanism, despite preventing the loss of the sulfido ligand during the reaction [145]. Overall, it still remains difficult to identify short‐lived reaction intermediates in the reaction of formate oxidation.

Another mechanism was proposed based on theoretical calculations involving selenocysteine dissociation from the Mo ion through a “sulfur shift” model [146, 147]. In this mechanism, Cerqueira, Gonzalez, Moura, and coworkers proposed that the oxidized enzyme is hexacoordinated by the two MGD molecules plus the selenocysteine and the terminal sulfido group (Figure 3A). When formate enters the active site, oriented by the positively charged arginine (e.g., Arg333 in *E. coli* FdhF), the repulsive environment generated would trigger a ligand shift to yield a Mo—S—SeCys moiety. This would create a vacant position to which the formate could bind. Subsequently, the S—SeCys bond is cleaved, and the selenol anion abstracts the formate C_α_ proton. Carbon dioxide is subsequently released. The catalytic cycle would be closed with the oxidation of Mo^IV^ to Mo^VI^ via intramolecular electron transfer to the [4Fe–4S] center and deprotonation of the selenocysteine residue. The role of the sulfido ligand, however, is also unclear in this mechanism [146, 147]. This mechanism can be applied to both formate dehydrogenase and nitrate reductase in a similar manner (Figure 3B). For nitrate reductase, however, an OAT mechanism has been accepted [2, 118, 148].

Hille and Niks proposed a mechanism that is based on experimental data [104, 123] and considers binding of formate to the second coordination sphere without contacting the Mo ion (Figure 3C) [149]. In their proposal, the sulfido ligand abstracts a hydride ion from formate, resulting in a two‐electron‐reduced intermediate Mo^IV^‐SH, and CO_2_ is released. In this mechanism, however, the (seleno‐)cysteine would not be involved, and the role of the metal ion is only to provide a hydride acceptor by coordinating the sulfido ligand. Therefore, recently, they revised their mechanism based on EXAFS HERFD studies [121]. They show that a persulfide is formed between the cysteine and the sulfur ligand, a state that resembles the first steps in the sulfur‐shift mechanism. The S—S bond is then cleaved to form an SH species, and the formal oxidation state of the molybdenum is not changed during the reaction. They suggest that the catalytic reaction of formate dehydrogenases involves ligand‐based redox chemistry at a metal center that is formally reduced to the Mo^IV^ state in the oxidized enzyme (Figure 3C, boxed inset) [121]. It is however difficult to agree with the oxidation state of the molybdenum in their study since in all previous studies, it was shown that the molybdenum active site is involved in the electron‐transfer reaction to the nearby [4Fe–4S] (also known as FS0) cluster and changes its oxidation state from VI to V and IV in a two and one electron‐transfer reaction. The persulfide formation would nevertheless be in agreement with other reaction mechanisms, like the OAT mechanism proposed for nitrate reductase (Figure 3).

Kumar et al. proposed an OAT mechanism in which the cysteine is displaced by water upon formate binding, that then transfers a hydride to the sulfido group and then results in the transfer of the hydroxo‐group from water to CO_2_, releasing bicarbonate, that further in the secondary reaction is converted to CO_2_ (Figure 3D) [145]. This mechanism can also be adapted to the new data of Hille and George, where the persulfide is formed upon formate binding, which opens a coordination site for water in this mechanism (Figure 3E). The persulfide is then reduced by hydride transfer, and in the further steps, the hydroxide is transferred to CO_2_, bicarbonate is released, and the six‐coordinate state of the cofactor is reformed after the release of bicarbonate with the intermediate Mo^V^ state with an SH‐group that was confirmed by EPR measurements [104]. This mechanism is supported by NMR data, EPR, and EXAFS data in addition to the inhibition by iodoacetamide and bicarbonate formation has been evidenced by NMR and labeling experiments [103, 104, 121, 138, 139, 145, 150].

As noted above, so far, none of these mechanisms have been unambiguously proven or accepted in the field, and some of them are highly under debate. Further investigations are, hence, urgently required to clarify the state of the sulfido ligand and its involvement in the mechanism. In a similar view, evidence for a bound sulfido ligand in periplasmic nitrate reductase and TMAO reductase persists, but in both cases, its presence currently depends on the methods for overexpression and purification (Table 2), and these differences affect the apparent view on participation of the active site sulfido ligand. In contrast to periplasmic nitrate reductase, TMAO reductase lacks an active‐site cysteine residue to partake in a sulfur‐shift mechanism. For molybdoenzymes lacking an active site Cys or SeCys ligand, the function of a bound sulfido ligand at the active site remains to be clarified.

For bis‐MGD enzymes such as FDH, the sulfido ligand contributes to their oxygen sensitivity since it is lost upon exposure to O_2_, resulting in an inactive enzyme with an oxido ligand present instead of a sulfido ligand (Figure 2B). This reaction is more pronounced for FDHs than for enzymes in the XO family, whose sensitivity to rather strong oxidation agents, superoxide and hydrogen peroxide, has been long known [67]. Unsurprisingly, as reported recently, FDH enzymes are also inactivated upon exposure to hydrogen peroxide and superoxide [75]. The presence of the inhibitor azide, however, stabilizes the sulfido ligand, suggesting that it binds in the vicinity of the sulfido ligand and prevents reactivity with O_2_ or other forms of ROS [105, 151]. Functionally, the coordination of the active site sulfido ligand in FDH enzymes versus, for example, an oxido ligand further impacts the redox potential of the bis‐MGD cofactor and its respective coupling to the FS0 [4Fe–4S] cluster, which may bias the catalytic reaction (Table 1) [3, 106]. Consequently, loss or lack of insertion of the sulfido ligand at the active site Mo or W ion typically renders the active site catalytically incompetent. The sulfido ligand is easily replaced by oxygen when oxygen is present in the uninhibited enzyme. The Hille group concluded that air inactivation is due to the production of superoxide by the *C. necator* enzyme in the presence of oxygen [74]. In this case, superoxide produced at the flavin mononucleotide (FMN) cofactor reacts with the sulfido ligand, which produces sulfite and replaces the sulfido ligand with oxygen. However, this mechanism of inactivation can only be applied to FMN‐containing enzymes that react with nicotinamide adenine dinucleotide (NAD^+^) or oxygen as electron acceptors, producing superoxide in the reaction with oxygen. Other FDH enzymes lacking a bound FMN must have a different mechanism, resulting in their oxygen/superoxide sensitivity.

For FDH enzymes, it was shown that upon loss of the sulfido ligand, it can be reinserted by a chemical resulfuration reaction similar to the one established for enzymes of the XO family more than 50 years ago [134] (Figure 2B). An in vitro sulfuration assay of *Rc*FDH was found to be rather O_2_‐sensitive, requiring strictly anaerobic and reducing conditions. Substantial incorporation of the sulfido ligand with sulfide was facilitated in the presence of the inhibitor azide, which itself protects the sulfido ligand from oxidative damage and inhibits FDH formate oxidation and CO_2_ reduction via binding in the second coordination sphere. Complete cofactor sulfuration with sulfide was possible in the presence of azide; however, strictly anaerobic buffer conditions were required. In addition, the presence of sulfur‐based reductants like dithionite or bisulfite in the presence of Eu^II^DTPA was also optimal and did not require the presence of azide [134]. Dithionite and bisulfite sulfur sources are also known O_2_ scavengers to produce bisulfite and/or bisulfate and have an additional positive effect on the reaction by removing O_2_ from the assay [152, 153, 154]. As explained above, it was shown previously for *C. necator* FDH that sulfite is produced upon superoxide oxidation of the active site sulfido ligand [74]. Such oxidation actually constitutes the reverse reaction to the transformation occurring during resulfuration and evidences the reversibility of the reaction. It can therefore be confirmed that sulfite may be also used as a sulfur source for the sulfido ligand at the bis‐MGD active site.

### 3.2 Identification of the Proteins Involved in XOR Moco Sulfuration

The concept of a common, molybdenum‐containing cofactor present in all molybdoproteins, with the exception of nitrogenases, was first proposed in the 1960s. Cove and Pateman [155] found that mutations in at least six different *Aspergillus nidulans* genes resulted in the simultaneous loss of XDH and nitrate reductase activities [155]. As these molybdoenzymes carry out completely different functions, the authors proposed that single mutations resulting in a loss of both these activities could identify genes somehow involved in the biosynthesis of a common cofactor. Mutations with a similar biochemical phenotype were also identified in *Drosophila*, bacteria, plants, and humans [156, 157, 158, 159]. The maroon‐like (*ma‐l*) mutations of *Drosophila melanogaster* [160] and the hypoxanthine nonutilizers, gene B (*hxB*) mutations of *Aspergillus nidulans,* identified a unique subset of cofactor mutations in that they lack some, but not all, molybdoenzyme activities. Mutations in *ma‐l* result in the loss of XDH, pyridoxal oxidase, and AO activities, but not SO activity [161]. hxB mutations result in the loss of XDH and nicotinate hydroxylase (purine hydroxylase II) activities but not nitrate reductase activity [162, 163]. Biochemical studies of both *ma‐l* [157, 161, 164, 165] and *hxB* mutant strains detected proteins corresponding to the missing activities, which have molecular weights, electrophoretic mobilities, and immunological properties identical to those in wild‐type strains. Enzymes of the XO family have a terminal sulfur atom covalently bound to molybdenum. This sulfur atom is not present in either nitrate reductase or SO, its place in these enzymes being taken by oxygen [1]. The studies identified that Ma‐l and HxB are involved in the sulfuration and therefore the activation of enzymes like XDH or AO in *Drosophila* and *Aspergillus* [161].

Both members of the XO family of Mo‐enzymes require a final step of maturation during or after insertion of Moco. These enzymes need the addition of a terminal inorganic sulfur to the Mo center in order to gain enzymatic activity. This sulfur ligand does not originate from the apoprotein, nor does it come from the Moco moiety. It was demonstrated that in vitro this sulfur can be spontaneously lost or can be removed from XDH/AO by cyanide treatment, generating an inactive enzyme [75] (as described above). The reaction, however, is reversible, and the enzyme can be reactivated by sulfide treatment under reducing conditions. In vivo, this terminal sulfur has to be added by a separate enzymatic reaction. In *Arabidopsis*, four AOs and XO lose their activity in an *aba3* mutant [166, 167]. In other eukaryotes, mutants similar to *aba3* are known as well. The genes for *ma‐l* (12) and for similar loci in humans (*hmcs*), cattle (*mcsu*), and fungi (*hxB*) were characterized [168, 169, 170, 171]. Sequence analyses of *Drosophila* and *Aspergillus* genes revealed homologies to bacterial NifS proteins, which are known as L‐cysteine desulfurases using pyridoxal phosphate (PLP) for transferring sulfur from L‐cysteine via a protein‐bound persulfide intermediate to various targets [170].

Bittner et al. identified the protein ABA3 from *A. thaliana* as a Moco‐sulfurase, catalyzing the insertion of the cyanolyzable sulfur into the active center of XDH and AO, thus leading to activation of these enzymes [166]. ABA3 is a two‐domain protein acting as a homodimer. Its N‐terminus shares significant homologies to bacterial cysteine desulfurases, thereby being more similar to SufS than to NifS or IscS [167, 172]. In a pyridoxal phosphate‐dependent manner, the N‐terminal domain of ABA3 decomposes L‐cysteine to yield alanine and elemental sulfur, the latter being bound as a persulfide to a highly conserved cysteine residue of ABA3. As the C‐terminal domain of ABA3 recently was found to efficiently bind sulfurated Moco [166, 172], it appears likely that the persulfide sulfur is transferred from the N‐terminal domain to Moco bound at the C‐terminal domain prior to the activation of the respective target enzyme. It is still unclear whether ABA3 finally activates its target enzymes by transferring only the sulfur, which is bound to the Moco of the C‐terminal domain, or whether it transfers the entire sulfurated Moco of the C‐terminus in order to activate XDH and AO [173]. In the first case, the C‐terminus thus would act as a scaffold for the assembly of a Mo—S center of which only the sulfur is subsequently passed to the Moco of the target enzymes. In the second case, the reaction catalyzed by ABA3 would be an exchange reaction where nonsulfurated Moco of the inactive target enzymes is replaced by sulfurated Moco from the C‐terminal domain of ABA3 [167, 172, 174].

Under physiological aspects, the terminal sulfuration step provides an efficient way of regulating the amount of active XDH and AO enzymes in the cell [171]. The concentration of physiologically active compounds like hormones, as produced by plant AO, and ROS, as produced by the action of AO and XDH from many species, can rapidly be increased by changing the ratio of inactive and active XDH and AO molecules. In fact, a rapid induction of the *aba3* gene was found upon drought and salt stress in *A. thaliana* as well as upon ABA treatment, thereby being consistent with the conditions associated with the requirements of activation of AO and XDH enzymes [175].

In contrast, in bacteria, it was shown that two proteins are necessary for the sulfuration of XDH enzymes, such as XdhC and NifS4 in *R. capsulatus*. XdhC thereby directly interacts with the Moco‐forming enzyme and receives the Moco straight from this enzyme [176]. Furthermore, it is believed that XdhC dissociates from this complex to interact with the L‐cysteine desulfurase NifS4. NifS4 interacts with XdhC and directly exchanges the equatorial oxygen ligand specifically for a sulfur ligand [177, 178]. The sulfur for this reaction originates from L‐cysteine, and a persulfide group is formed on NifS4 in the course of the reaction [176]. Furthermore, XdhC has to dissociate from NifS4 after the sulfuration reaction to interact with XdhB, the Moco‐containing subunit of XDH [177]. The results for *R. capsulatus* XDH demonstrate that dimerization via the β‐subunits is required to stabilize the structure of the protein that makes the protein suitable for Moco insertion. Since XdhC is a dimer itself, it is suggested that the XdhC dimer interacts with the XdhB dimer simultaneously, inserting matured and sulfurated Mo‐MPT into both active sites of XdhB [177, 179, 180]. In conclusion, the sulfuration of Moco is different in bacteria and eukaryotes since so far, no in vivo repair of desulfo enzymes could be identified in bacteria. If this mechanism indeed exists in eukaryotes, it remains to be determined.

### 3.3 Identification of the Protein Responsible for Sulfuration of FDH Enzymes

In *E. coli*, the FDH activity requires the protein FdhD that was reported to act as a sulfurtransferase between the L‐cysteine desulfurase IscS and FDHs for the insertion of the sulfido ligand in these enzymes [181, 182].

*E. coli* synthesizes three FDHs that decompose formate into carbon dioxide and protons. FdnGHI (the so‐called FDH‐N) and FdoGHI (FDH‐O) are respiratory enzymes anchored to the periplasmic side of the inner membrane [183, 184, 185]. FdhF (FDH‐H) is part of the fermentative formate–hydrogen lyase complex and is located at the cytoplasmic side of the membrane [186]. All three FDHs share a common requirement for FdhD for their activity [187]. Residues Cys121 and Cys124 of *Ec*FdhD have been suggested to be involved in the sulfurtransferase reaction between L‐cysteine desulfurase IscS and FDHs [182]. The current working model predicts that Cys121 is the catalytic residue involved in both sulfur transfer from IscS to *Ec*FdhD and FDH activation, while Cys124 of *Ec*FdhD promotes sulfur release from the persulfurated Cys121 residue to the terminal sulfur acceptor, bis‐MGD; *Ec*FdhD likely possesses a sophisticated mechanism to ensure modification of bis‐MGD at the correct position [182]. However, the detailed catalytic mechanism of molybdenum cofactor sulfuration in FDHs, as well as AO or XOR and periplasmic nitrate reductases, is still unknown [26]. The question has been difficult to address because the molybdenum cofactor is oxygen sensitive, very labile, and never found in its free state in organisms. Moreover, three‐dimensional (3D) structures of molybdoenzymes demonstrate that it is systematically found buried in the enzyme [188]. Combining the structural basis and the suggested sulfurtransferase activity of *Ec*FdhD, a mechanism by which the sulfur is transported from IscS on one side of the FdhD dimer to the molybdenum cofactor on the other side prior to its final insertion into FDH has been suggested [182]. The mechanism still needs to be proven, though.

### 3.4 Evolutionary and Regulatory Aspects

FDH enzymes must have been among the earliest molybdoenzymes already present in LUCA [189] since phylogenetic analyses demonstrated that formate oxidation and CO_2_ reduction were the ancestral functions of the DMSO reductase superfamily [190, 191]. Conclusively, the sulfido ligand mainly present in FDH enzymes of the DMSO reductase family was lost during the evolution of the other members of this family, likely based on its oxygen sensitivity when oxygen became available. New functions of the enzymes then evolved independent of the sulfido ligand. Exceptions, however, are nitrate reductase and TMAO reductase, which likely still retained the sulfido ligand. Nitrate reductase has an almost identical structure to FDH enzymes with the same first coordination sphere containing a sulfido ligand and a cysteine [192]. While the sulfido ligand is under debate for some nitrate reductase enzymes, it has been proven crystallographically for the *C. necator* enzyme [121]. The enzyme has likely evolved for a different substrate specificity, taking nitrate instead of formate as a substrate and has evolved further toward an OAT mechanism in which the sulfido ligand is not actively involved. To be active with an oxido ligand for nitrate reduction might have been advantageous based on the labile nature of the sulfido ligand toward oxygen. Later, a regulatory mechanism has evolved, expressing the enzymes only in the absence of oxygen under the control of the transcriptional regulator FNR [193]. FNR is a Fe–S cluster‐dependent transcriptional dual regulator, an important transcription factor involved in the regulation of genes with a role in anaerobic respiration, which senses the availability of oxygen via the functionality of a [4Fe–4S] cluster that is converted to a [2Fe–2S] cluster in the presence of oxygen [194]. Only *E. coli* TMAO reductase, expressed by the TorCAD operon, escapes the FNR regulation and is also expressed under aerobic conditions [195]. Here, another mechanism is present to maintain TMAO reduction in the presence of oxygen [125]. While the enzyme is rapidly inactivated when oxygen becomes available, likely based on the loss of the sulfido ligand, a bet‐hedging mechanism has evolved, producing more enzyme when oxygen is present probably to replace the inactivated enzyme [196, 197]. By this mechanism, the organism sacrifices an enzyme likely to be flexible under continuously changing conditions in growing cultures [197]. Since the presence of the sulfido ligand is not accepted yet in this enzyme and it is not actively involved in the OAT mechanism of TMAO reduction, its role might also be to trigger the redox potential of the enzyme and to change its substrate specificity toward other substrates that also are available under the specific culture conditions [125]. This is completely hypothetical and needs to be investigated in future studies.

## 4 The Oxygen Sensitivity of the Sulfido Ligand From a Chemical Perspective

During the course of over 100 years of work on Moco, it has emerged that there is a notable and consistent difference between two enzyme families with regard to the stability or lability of their sulfido‐bearing active sites. The XO family of enzymes coordinates a sulfido ligand that is relatively stable against molecular oxygen, whereas the DMSO reductase family of enzymes coordinates a sulfido ligand that is highly labile in the presence of oxygen species (see above). Exceptions to the latter are known among characterized FDHs, which in themselves constitute a focus within the field and comprise aspects external to the active site that are not conserved across characterized FDHs, thereby falling outside the scope of this review [198]. Structurally, the most prominent distinction between the two families addressed here is the number of coordinated MPT ligands, besides the presence or absence of various amino acid ligands (Figure 1). Those enzymatic active sites with a single MPT ligand (as in XO) will be subsequently referred to as mono‐MPT and those with two (as in DMSOR) as bis‐MPT for the sake of simplicity in the discussion.

The active sites are buried deep inside a protein framework, which inter alia stabilizes the MPT ligand(s) via hydrogen‐bonding interactions [2, 199]. The placement of the prosthetic group inside its designated protein pocket prevents unwanted side reactivity, as observed in catalytically investigated synthetic model complexes, including catalyst dimerization and destructive ligand oxidation [200]. On this basis, the chemical structure of the prosthetic group in the enzyme itself is a predominant defining factor for O_2_ stability/lability of the sulfido ligand.

The following contemplations about the probable reasons for the chemical difference in stability between mono‐MPT versus bis‐MPT active sites are based on established facts, but it is speculative to some extent, while we believe it to be overall reasonable. Observations, which form the basis of the subsequent discussion, are the following:

i. Mono‐MPT active sites undergo sulfido ligand replacement by an oxido ligand in the presence of superoxide or peroxide only in their reduced oxidation state but not in the oxidized oxidation state.
ii. Bis‐MPT active sites are very labile with regard to sulfido‐to‐oxido exchange in any oxidation state already in the presence of merely molecular oxygen and also very sensitive to superoxide and peroxide.

The redox‐dependent sensitivity of a mono‐MPT site only to undergo sulfido‐to‐oxido exchange upon reduction suggests that lability is associated with an increase in electron density at molybdenum and/or the sulfido ligand, which in the reduced enzyme form also bears a proton forming a Mo—SH moiety (see above). By accepting the proton (regardless of whether “H” is introduced as a hydride with concomitant relay of two electrons to molybdenum or a different electron movement takes place), the sulfur formally buffers electron density away from the electron‐rich active site metal center. In the reduced state, not only the molybdenum ion is more electron density rich but the sulfur is as well. Bearing more electron density in relation results in a redox potential being relatively lower (less positive/more negative), and the respective atom is easier to be oxidized. A direct attack of an ROS on the sulfur atom in the reduced state due to its relatively higher electron density renders it more vulnerable to being oxidized by an electronically unsaturated oxygen species compared to an oxidized active site, in which the sulfur pushes more electron density toward the coordinated molybdenum. This is reflected to some extent in the reassessed relation between the “very rapid” (featuring a Mo=S bond) and the “rapid” Mo^V^ species of XO, the latter of which having been found to represent a nonproductive complex with the partially reduced enzyme harboring a Mo^V^ oxidation state with a Mo—SH bond [201, 202]. In a reduced state, the sulfido ligand is generally less Lewis basic to molybdenum, hence less strongly bonded to it and consequently more easily ousted by O^2−^ originating from the ROS (superoxide or peroxide) after its oxidation of the sulfur or from water.

Ascertaining a potential difference in stability between a sulfido species compared to an oxido species in the case of an oxidation event that formally introduces an oxygen atom is challenging in model chemistry and in general since an oxido species is already the outcome of such a reaction, and it can also result in a coordination geometry change at the metal. Notably, the Sugimoto group investigated atom transfer in the catalytic half‐reaction from the metal to a phosphine substrate [203]. In some metal complex species with tungsten(VI) or molybdenum(VI) centers, in which both an oxido and a sulfido ligand were present, exclusively sulfur was transferred in stoichiometric reactions as well as with phosphine excess. The observation implies that (at least in structurally similar model compounds for the Moco‐dependent enzymes) the Mo=S bond is slightly more labile and/or the resulting P=S species kinetically or thermodynamically more favorable. We have in our own research never encountered any observations that would be in disagreement with these findings of the Sugimoto group.

It was noted before by the same group that the impact which an apical chalcogenide ligand has on the redox potential of a model complex of the type [MoCh(dt)_2_]^2−^ (Ch = O, S, Se; dt = dithiolene) is negligible compared to the influence of the nature of the dithiolene ligands [204]. A literature survey of the available pairs of analogous Mo^IV^O and Mo^IV^S complexes with at least one dithiolene ligand and reported redox potentials for their one‐electron oxidation confirms this (Table 3). The largest observed difference in potentials was 90 mV, and the most common difference is 30–50 mV, whereas differences associated with distinct dithiolene ligands were found to be approximately ~300 mV [204]. This implies also that a replacement of a sulfido by an oxido ligand in Moco‐dependent enzymes does not substantially affect the general redox stability of the prosthetic group.

**TABLE 3:** Redox potentials in V (vs. SCE) of analogous pairs of Mo=S and Mo=O complexes with at least one dithiolene ligand, and their differences in mV.

Chemical structure of [Mo(S/O)(dt)_1/2_L_0/1_]^2−^	*E*_1/2_ (MoS)	*E*_1/2_ (MoO)	Δ*E*_1/2_	Reference(s)
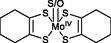	−0.75	−0.70	50	[204]
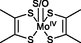	−0.65	−0.62	30	[205, 206]
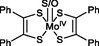	−0.43^a^	−0.46^a^	30	[204]
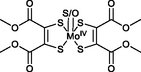	−0.12	−0.03	90	[204]
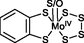	−0.10	−0.05	50	[207]
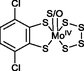	+0.04	+0.05	10	[207]

a It is the only pair in which the oxido complex has a lower potential than the sulfido complex. This may be correlated to their cyclic voltammograms not being electrochemically reversible and the data potentially not entirely reliable. In the five other pairs, the sulfido complex is consistently easier to oxidize than the oxido complex, which appears chemically reasonable.

In the enzymes, the number of coordinated MPT ligands is, hence, apparently highly relevant for stablilizing a coordinated terminal sulfido ligand. A notable feature of ene‐1,2‐dithiolate (or dithiolene) ligand systems in general, a group to which MPT belongs, is their redox noninnocence [208]. This means that the ligand is in principle able to push entire electrons onto their coordinated metal center while being oxidized themselves to a radical form or to a dithione if they pass on two electrons (Figure 4a) [209]. For mononuclear synthetic model complexes, a small number of examples of actual dithione molybdenum complexes have been reported [210, 211, 212, 213, 214, 215, 216, 217, 218, 219].

**FIGURE 4:**
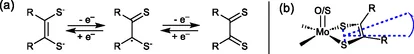
(a) The ability of dithiolene ligands to be oxidized by one or two electrons, which could take place as complex‐internal redox reaction. (b) The folding angle which may be used to quantify the effect of increased π‐donation from sulfur to metal.

Quite notably, all respective oxidized versions of the ligand system bear amine nitrogen functions adjacent to the (ene‐dithiolate‐turned) *cis*‐dithione moiety. The lone pair on nitrogen is likely extensively delocalized toward the two carbon and two sulfur atoms to facilitate some stabilizing resonance occurring via an extended π‐system in support of keeping the ligands oxidized. Molybdenum complexes with radical anion forms are exceptionally rare, with only a single example of a quite notable crystallographically characterized complex system [220]. The central Mo^V^ bears two ligands with an apparently nearly identical structure, while one is in the dithiolene form and the second is in the radical anion form as their only difference. The ligands employed here also carry an amine nitrogen adjacent to the C_2_S_2_ unit. It must be pointed out, though, that in the enzymes, no such adjacent *N* functions are present and, consequently, the radical anion or extreme dithione forms have to date not been found on MPT, even if they may provide partial contributions to the overall electronic structures, in particular in the oxidation state Mo^V^ of Moco through electron density delocalization between MPT and Mo.

Potentially more importantly, dithiolene ligands, and this includes at least to some extent the molybdenum cofactor's MPT ligands, are excellent at donating electron density to the metal not only as σ‐donors but as π‐donors as well. The latter can be accomplished or increased by a bend along the S‐‐‐S line (resulting in an angle between the M—S—S plane and the S—C=C—S plane deviating from 180°; Figure 4b) that brings the p orbitals of the sulfur atoms in which free electron pairs reside closer to the coordinated metal's orbitals facilitating overlap [221, 222]. The geometric result of this motion was defined as the folding angle by which the geometric effect can be quantified. Dithiolene ligands (likely including MPT) can therefore provide more electron density to their coordinated metal ion than normal ligands can, and this may be controlled with the folding angle adapted, which increases with the oxidation state [213].

In a computational study of bis‐dithiolene model complexes, it was shown that the two ligands behave differently but in synergy, which likely increases the overall push of electron density to the central metal [223]. The donation of electron density, in fact, becomes so strong that the dithiolene sulfur atoms adopt positive natural population analysis (NPA) charges in the highest formal oxidation state of the metal (Mo^VI^), while the central metal is even less positively NPA charged than in oxidation state +V. Unfortunately, such a systematically focused computational study is not (yet) available for comparable mono‐dithiolene model complexes. However, it appears reasonable that in the absence of the second dithiolene ligand, the effect will be less pronounced. In summary, various observations suggest that with two MPT present in the active site, more electron density can be donated to the central metal in any oxidation state compared to the mono‐MPT active sites, that with more electron density on the metal, the sulfido ligand is also electron density richer than in the mono‐MPT species, and that this makes it easier for an electrophilic oxidation agent to take an electron (or two) away from the sulfido ligand sulfur. An oxidized S is less Lewis acidic and can, hence, be easily displaced by an oxido ligand from molecular oxygen or any ROS that took up the electron from the sulfur atom or after its oxidative removal by an oxido ligand stemming from water. This would be in agreement with the observed better stability of mono‐MPT versus bis‐MPT active sites and might be a reasonable explanation for this phenomenon.

Regardless of the exact mechanism of sulfido loss described herein, in all above discussed scenarios, it will be generally more feasible to transfer an electron onto the attacking oxygen species if there is more electron density already residing on the Mo=S moiety and if the loss of electrons can be more efficiently buffered by the remaining ligands. For both these features, the presence of two rather than only one MPT ligand will be more beneficial toward promoting sulfido ligand loss. The extent of the push of electron density or electrons onto the active site molybdenum is reasonably considered stronger with two MPT ligands, which likely results in an increased vulnerability for an electrophilic attack by an oxygen species. This also can cause additional oxidation/damage of the cofactor, resulting in the loss of coordination of a MPT ligand at the metal, as has been observed experimentally for DMSO reductase [93, 96, 97, 98] and TMAO reductase [125]. In conclusion, we believe the oxidizability is increased in the bis‐MPT case compared to the mono‐MPT case and that this is the predominant reason for the observed differences in stability rather than distinct bond stabilities between molybdenum and the sulfido ligand in mono‐MPT versus bis‐MPT.

## 5 Summary and Outlook

The past 125 years have provided considerable insight toward the role played by the terminal sulfido ligand bound to mononuclear molybdo‐ and tungstoenzymes. XO has been studied for more than 100 years, the enzyme was comprehensively characterized, and its hydroxylation mechanism is well accepted in the field. The role of the sulfido ligand in XOR has been established to act as a hydride acceptor during catalysis. On the other hand, metal‐containing formate dehydrogenases are an emerging enzyme to be studied in the field of molybdo‐ and tungstoenzymes based on their ability to catalyze the reaction of CO_2_ reduction. This enzyme was the first known to bind a sulfido ligand in the first coordination sphere in enzymes of the DMSO reductase family, which was initially not evident from the first crystal structure of *E. coli* FdhF and was revised by later investigations. Subsequent studies pointed to a role of the sulfido ligand in FDH as a hydride acceptor from the substrate formate, producing directly CO_2_. More recently, the presence of the sulfido ligand was revised, with the sulfur in the oxidized enzyme merely being part of a persulfide together with the nearby cysteine, putting the former studies into question. Further, the reaction mechanism of FDH enzymes is still under debate whether it is an OAT mechanism or a mechanism taking place exclusively in the second coordination sphere without any direct involvement of the metal ion. Notably, a sulfido ligand was also found in other enzymes of the DMSO reductase family, that is, in nitrate reductase and TMAO reductase. The presence of this ligand and whether it is essential still have to be unambiguously established in these enzymes. The difficulty of the identification of the sulfido ligand in purified enzymes of the DMSO reductase family is clearly its oxygen sensitivity since it is easily lost when the enzyme is not purified under strictly anaerobic conditions. In conclusion, there are many research questions left on the characterization of these enzymes, in particular with regard to clarifying the presence and role of the sulfido ligand in their catalytic mechanisms. We consider this to be one of the most urgent tasks for everyone engaged in this fascinating research area.

## Funding

This study was supported by Deutsche Forschungsgemeinschaft (SPP1927).

## Conflicts of Interest

The authors declare no conflicts of interest.

## Data Availability

The data that support the findings of this study are available on request from the corresponding author. The data are not publicly available due to privacy or ethical restrictions.
